# Research with adolescents who engage in non-suicidal self-injury: ethical considerations and challenges

**DOI:** 10.1186/s13034-015-0071-6

**Published:** 2015-09-28

**Authors:** Elizabeth E Lloyd-Richardson, Stephen P Lewis, Janis L Whitlock, Karen Rodham, Heather T Schatten

**Affiliations:** Department of Psychology, University of Massachusetts Dartmouth, 285 Old Westport Road, North Dartmouth, MA 02747 USA; University of Guelph, Guelph, Canada; Bronfenbrenner Center for Translational Research, Cornell University, Ithaca, NY USA; Staffordshire University, Stoke-on-Trent, UK; Butler Hospital, Alpert Medical School, Brown University, Providence, RI USA; Department of Psychiatry and Human Behavior, Alpert Medical School, Brown University, Providence, RI USA

**Keywords:** Ethics, Non-suicidal self-injury, Self-harm, Adolescence, Imminent risk, Risk assessment, Research

## Abstract

Non-suicidal self-injury (NSSI) has emerged as a significant psychiatric issue among youth. In addition to its high prevalence rates, NSSI is associated with a number of psychiatric issues and confers risk for varying degrees of physical injury. It is also a risk factor for attempted suicide. Thus, youth who engage in NSSI represent a vulnerable and high-risk population and researchers are likely to encounter a variety of ethical challenges when conducting NSSI research. Accordingly, it is critical that researchers be familiar with the major ethical issues involved in NSSI research and how to effectively account for and address them. This is important both prior to obtaining clearance from their Institutional Review Boards and when carrying out their research. To date, there is no consolidated resource to delineate the ethical challenges inherent to NSSI research and how these can be effectively navigated throughout the research process. The goals of this paper are to review international best practices in NSSI research across the various contexts within which it is studied, to offer guidelines for managing these issues, to identify areas in which variation in approaches prohibits decisive recommendations, and to generate questions in need of further consideration among scholars in this field.

## Background

Non-suicidal self-injury (NSSI) is the deliberate, self-inflicted destruction of body tissue (e.g., cutting, burning) without suicidal intent and for purposes not socially sanctioned. NSSI is included in the fifth edition of the *Diagnostic and Statistical Manual of Mental Disorders* as a condition requiring further research before consideration as an official diagnosis [1]. The proposed criteria require NSSI incidents on five or more days within the past year, with at least one of the following expectations: to seek relief from a negative feeling or cognitive state, to resolve an interpersonal difficulty, or to induce a positive state. The behavior must also be associated with one of the following: interpersonal difficulty or negative feelings and thoughts (e.g., depression, anxiety), premeditation, and/or ruminating on NSSI. Scab picking, nail biting, and socially sanctioned behaviors like body piercing and tattooing, do not qualify for the diagnosis.

Although not a new phenomenon, NSSI prevalence among adolescents and young adults is high and broadly distributed across both community and psychiatric samples. The comorbidity and consequences of NSSI are significant. It is a strong risk factor for suicide and is associated with a host of psychological difficulties and disorders which include, but are not limited to: mood and anxiety disorders, borderline personality disorder, substance abuse, difficulties with negative affect (e.g., anxiety, frustration), hopelessness, self-criticism, poor body image, and low self-esteem [2, 3].

Youth who self-injure are generally considered a vulnerable population, both because of the potential for unanticipated injury in the course of self-injuring and because of the possible presence of other serious comorbid issues, such as suicidal thoughts and behaviors. Balancing the need for both a clinical and public health understanding of the phenomenon with the individual need for privacy and safety can produce ethical issues and dilemmas for researchers, study participants, and clinicians.

Knowledge of the moral principles and enforceable standards underlying the ethical conduct of research with human participants is important to researchers because “merely following the requirements of law, federal regulators, ethics committees and IRBs [Institutional Review Boards] does not absolve the researcher from personal responsibility for resolving possible ethical conflicts that may arise in the conduct of their work” (p. 9) [4]. The 1949 Nuremberg Code and subsequent 1964 Declaration of Helsinki firmly establish that researchers and medical professionals should do no harm in their practice and research [5].

With this background in mind, the primary goal of this paper is to review international best practices in NSSI research across the various contexts within which it is studied. Currently, NSSI research is conducted in a variety of settings, some of which impose constraints on capacity to assess and respond to imminent risk and possible iatrogenic effects. For example, collecting data via web-based surveys or interventions may likely prohibit the same level of assessment and response as is possible in face-to-face interviews. Similarly, interviews conducted via phone or Skype will not permit the same level of assessment and response as is possible with in-person visits occurring in a lab or clinical setting. Add to this broader institutional considerations, such as liability, which may be accrued to an institution for not being able to immediately respond to knowledge of imminent risk (such as may occur in web-based survey research where responses enter a database which may not be accessed for weeks), and best practices for assuring that the needs of researchers, participants, and institutions are met can become very unclear.

Balancing participant needs and protections with researcher aims and the constraints imposed by the research setting necessitates consideration of a broad range of issues, including: consent and assent, privacy, confidentiality, and imminent risk, risk assessment and response (including the decision to intervene), iatrogenic effects, professional competency and overall safety for participants *and* researchers, and benefits to participants. Throughout this paper, face-to-face contexts and settings involving direct research contact are distinguished from remote or web-based contexts (e.g., large scale assessment studies, random-digit dialing, Internet forums) that, by their nature, do not involve direct contact and can be anonymous. Where considerations differ by study context, it is noted, thus highlighting the intense variability and consideration that must be taken into account, including the intent and scope of the study, the research context, and the expectations that the local IRB and research participants themselves might have.

Although NSSI research is now developed enough to highlight commonalities in research approaches and ethical issues encountered, there remain a variety of areas in which established researchers apply different criteria or processes, especially when it comes to assessing risk and breaching confidentiality. Because of this, in addition to the guidelines and recommendations included here, we identify areas in which variation in approaches prohibits decisive recommendations, and identify questions in need of consideration among scholars in this field.

## Review

### Issues associated with consent and assent

“Informed consent” is the voluntary agreement of an individual, or an authorized representative, who is not provided undue inducement nor otherwise coerced to participate in research. Only individuals who have reached the legal age of consent can provide consent, which varies by region (e.g., in the U.S. this is typically 18 years old). “Assent” is the agreement of someone not able to give legal consent to participate in the activity. The individual must possess adequate knowledge and understanding of the proposed research, the risks and potential benefits, and the importance of making an informed decision [6]. If assent is provided, informed consent must still be obtained from the individual’s parents or guardian unless obtaining consent poses no more than minimal risk to the children, would not adversely affect the rights and welfare of children if a waiver of consent is approved, or if research could not be carried out without the waiver. In the U.S., waivers are granted only after conferral with IRBs and are typically rare.

Parental consent can be “passive” or “active” and when conducting research with youth, deciding whether to obtain active versus passive consent represents an important ethical consideration. The type of consent sought by the researchers can significantly affect participation rates, study costs, selection biases, and thus, sample representativeness [7]. Passive consent assumes that a non-response from a parent/guardian indicates latent consent (i.e., permission has been granted for the young person to take part in the research). In contrast, when active consent is sought, written consent is required and a non-response indicates an absence of parental/guardian consent. In general, passive consent is often preferable to researchers because it enhances the likelihood of more robust youth participation. In most cases, ethical standards and IRBs will require or encourage active consent, even while recognizing that parental permission is not always a reasonable requirement for research with adolescents because of the need to protect the autonomy and privacy of youth when the nature of the subject being investigated is particularly sensitive [8, 9]. Therefore, researchers need to carefully consider and balance caregiver desires and concerns about their child’s vulnerability with their child’s capacity to make autonomous decisions about his/her participation. Readers are encouraged to consult their own country’s rules, regulations, and norms.

In research involving direct contact (i.e., face-to-face, visual and/or auditory), research team members responsible for obtaining informed consent should be fully aware of the study protocol, and be trained to ensure that the participant fully understands what is involved and is given ample time to discuss questions and/or concerns. In cases in which there is no direct participant contact (e.g., online studies, large-scale research studies), participants should be encouraged to contact the researchers about any study-related questions they may have; appropriate contact information should therefore be included in the consent document. In all research settings, research team members should remind participants and their parents/guardians that they have the right to withdraw from study participation even if they have previously given consent or assent.

### Confidentiality, privacy, and disclosure of imminent risk involving youth

Most professional mental health organizations’ by-laws, as well as country-specific state or provincial laws, detail the exceptional circumstances when confidentiality may need to be broken. In other words, circumstances under which there appears sufficient evidence to raise serious concern about the: safety of clients; safety of other persons who may be endangered by the client’s behavior; health, welfare, or safety of children and vulnerable adults; unethical and illegal conduct (e.g., abuse) by health professionals. It is incumbent upon researchers to clearly outline the limits to confidentiality pertinent to their jurisdiction and profession at the outset of study participation so that young people and their parents are aware of all limitations to privacy and know what to expect in these circumstances. As with the discussion above, clearly these limitations will be shaped by the type of research context and level of contact with participants.

In remote or web-based studies collecting anonymous data, informed consent and assent documents must clearly outline the value that anonymity and confidentiality offer, but also remind participants and parents/guardians that imminent risk cannot always be adequately assessed or addressed. In research settings involving face-to-face contact, and perhaps including audio or video contact as well, informed consent and assent documents should clearly state circumstances under which confidentiality between researchers and participants will be breached or cannot be maintained. In the case of studies located in clinical research settings, such as in academic medical centers, it is critical that researchers make clear their role as a researcher, versus a clinician, with participants before interacting with them. As Prinstein and Helms [10] point out, a clinical interview aims to assess psychological functioning, help patients to discover information about themselves, and to determine the next steps in treatment. A research interview generally uses a structured approach to gathering information with the intention of generating new information that will be applied to a larger sample or population. In the participant’s eyes, however, the methods used in both of these cases would appear quite similar: both interviewing techniques aim to build rapport, and both ask many questions with the aim of gathering information.

Although there is some variability across IRBs, there is often a mandate stipulating that research procedures may never be allowed to interfere with clinical work, nor shall research records and clinical medical records be allowed to intermix. While this serves to protect patient privacy and confidentiality, it can lead participants and their family members to feel confusion over an apparent lack of communication between their care providers. This can be managed by ensuring that participant information sheets are absolutely clear that the research is separate from any care or treatment the participant might be experiencing and that the only time their caregivers may be informed of what the participant mentions is if the participant were to disclose something that suggests they might be at risk of suicide, harm to others, or experiencing abuse. This also requires that researchers clearly assure participants that engaging in research has absolutely no impact on participant ability to receive clinical care or on the quality of this care. While this may seem straightforward, effectively understanding, anticipating and addressing confusion visible to the participant but not the researcher is an orientation in which few researchers are trained. Preparing researchers to understand from the participant perspective and to communicate effectively and in an assuring manner through role-play can be beneficial in correcting participants’ false impressions.

Why does this become important from the perspective of privacy and confidentiality? If the distinction is not made clear from the start, participants may disclose personal information (such as suicidal intention) and may be dismayed when they learn that this information must be reported because of ethical or legal requirements imposed on researchers in ways that differ perhaps from their clinician confidantes or other online surveys they may have participated in. Readers may also wish to consult Miller, Rathus and Linehan [11] for detailed information about managing confidentiality issues when conducting research in clinical contexts with youth at risk for NSSI and suicide and their families.

Prinstein and Helms [10] provide sample consent and assent language that would be useful in face-to-face contexts that do not involve anonymity. Modified to differentiate risk of suicide, as opposed to more generalized harm (e.g., NSSI, substance abuse), an example of consent wording is as follows:

*…the Certificate of Confidentiality*^a^*does not prevent the investigator from taking necessary action to protect participants or others from harm in certain situations. We may contact you and/or proper authorities (e.g., your child’s therapist, Child Protective Services, the police, emergency mental health services) if your child reports suicidality, threatens severe harm to others, or discloses information about suspected or known sexual, physical, or other abuse. If any member of the research team is given such information, he or she will make a report to the appropriate authorities.*

An example of assent wording is as follows:

*All information we collect from you will be kept entirely confidential (secret). Your parents, teacher, and school will NOT have access to the information obtained from you…There are exceptions to these rules of confidentiality: If you tell us that you may be in serious danger or risk of suicide or ending your life, harming someone else, or if you provide information about sexual, physical, or other abuse that you may have experienced…we will contact the proper authorities to make sure that you are safe.*

### Clarifying privacy and disclosure of imminent risk with the IRB

The following language may serve as a starting point for describing to an IRB the specific parameters of imminent risk for face-to-face studies, which should then be followed by how that particular study’s risk assessment protocol will manage evaluation and handling of these cases:

*We define “imminent risk of self*-*injury” as a strong likelihood that an adolescent will engage in life threatening, self*-*injurious behavior within 48* *h of our assessment. Unfortunately, no algorithm is available to determine in a reliable and valid way whether someone is likely to engage in life*-*threatening behavior within 48* *h. However, in our past research we have developed a detailed protocol that allows us to identify and intervene in a cautious and safe manner* [10].

The following language may assist in describing to an IRB the parameters of imminent risk for remote and/or web-based studies, when researchers are not in a position to respond with a detailed risk assessment protocol. While the use of “distract buttons” is further discussed later, we recommend incorporating them as a simple and direct strategy for allaying concerns the IRB may still have despite a lack of empirical evidence of NSSI questions leading to iatrogenic effects:

*Since we will not be conducting interviews, there will be no way for us to know whether someone is experiencing extreme duress. Although the survey contains questions designed to detect duress at some point in life, none of the questions are time sensitive enough to permit us to know whether they are experiencing distress during the survey. To reduce any risks posed by the survey, participants will be alerted to the risks in the survey, encouraged to discontinue the survey at any time they become uncomfortable, provided with a “distract button” on every page where participants can effectively take a break from the questions by being routed quickly to a neutral news page, and provided a list of local mental health resources with activated web links at the conclusion of the survey. To assure that they have needed resources as the survey progresses, a link to [university health services] webpage, the phone number for the 24*-*hour crisis line, an e*-*mail for the study coordinator or director. We will also insert text at the beginning of the section which starts the series on self*-*injury to inform respondents that they will be asked a series of NSSI*-*related questions and that resources links are provided at the bottom of each page to assist them if they want or need to talk to someone.*

### Weighing the balance of privacy, confidentiality and imminent risk

As discussed previously, researchers are ethically obligated to report imminent risk of life-threatening self-harm in certain research contexts. However, determining which behaviors to consider life-threatening and what time period to consider imminent raises many complex questions. Before researchers can establish when to respond to imminent risk, and therefore break confidentiality, it is necessary to clarify and define imminent risk. Below follows a discussion devoted more specifically to face-to-face contexts and settings involved direct research contact, and the heightened consideration of risk assessment that researchers must carefully evaluate.

#### Clarifying and defining imminent risk and self-harm behaviors

Imminent risk is often discussed in the context of suicide, where definitions of imminent risk vary (e.g., next 48 h, next 7 days), and suicide risk can vary from moment to moment [12]. How do researchers determine imminent risk when someone reports NSSI? By definition, NSSI involves a lack of intent to die; yet, NSSI is a risk factor for suicidal thoughts and behaviors. In addition, by its very nature (e.g., cutting), NSSI may have unintended but possibly lethal consequences. Research in this area brings up numerous questions with regard to whether or not researchers are ethically obligated to break confidentiality when an adolescent discloses that they are engaging in NSSI. In the following section, we discuss imminent risk and potential risk factors that may point to the need for a more thorough risk assessment.

#### Differentiating suicidal and non-suicidal thoughts and behavior

As discussed above, adolescents and young adults who report a history of NSSI may also report experiencing suicidal thoughts and behaviors [13–16]. In face-to-face research, which may even include multiple meetings with a particular research participant, some researchers argue that it is imperative that a competent imminent risk assessment is completed, paying particular attention to “red flag” warnings of suicidal ideation and behavior as well as NSSI.

Although both NSSI and attempted suicide involve deliberate harm to the body, and often co-occur, these behaviors differ in suicidal intent, perception of the event, proposed function of the behavior, chronicity, and method [17–19]. For example, NSSI tends to be a chronic and repetitive behavior while suicide attempts occur more infrequently, and injuries from NSSI are usually of lower lethality than injuries from attempted suicide [18]. However, it is important to note that NSSI may increase in risk and lethality over time [20]. Therefore, although NSSI is performed with no intent to die, it is possible that self-injurious behavior could lead to major injury or even unintentional death.

Ostensibly, more severe injuries, especially those meriting medical attention, would indicate a higher level of risk. Hence, these instances may warrant that confidentiality be breached. Complicating this, however, are several factors. First, there are no clear guidelines on how to assess the medical severity of NSSI injuries. Second, many NSSI researchers do not have the requisite medical training to properly assess the nature of injuries. Third, participants may find questions about or requests to show injuries (for the purpose of assessment) to be invasive, especially if there is no existing therapeutic relationship with the researcher. As the field continues to grow, it will be important to consider these issues in order to determine how to best manage and understand risk among individuals who self-injure.

### NSSI and risk assessment protocols

#### NSSI assessment tools

Simply asking about NSSI may result in ambiguous situations in terms of the nature of behaviors reported (e.g., severity, potential for lethality) and one’s corresponding duty to report. As discussed below, to accurately determine the nature of NSSI behavior engaged in by young research participants, the use of empirically validated measures can be helpful. There are multiple tools available to aid researchers in the identification of suicidal and non-suicidal thoughts and behaviors, including self-report measures and structured and semi-structured interviews, which vary in breadth and number of items. Table 1 lists recommended assessment measures for these constructs. Detailed review of the psychometric properties of each, as well as discussion of clinical utility, is provided elsewhere [21]. It is important to note, however, that the purpose of these tools is to gather reliable and valid data, and that they are designed to give a crude indication of the level of potential risk of harm. Although these measures may indicate potential “red flags” and can guide risk assessment, they should not be used to predict future suicide or risk for life-threatening self-harm in and of themselves.

**Table 1:** Assessment of suicidal and non-suicidal thoughts and behaviors

Assessment measure	Assessment focus
*Self-report measures*	
Functional Assessment of Self-Mutilation (FASM) [58]	NSSI history, methods, frequency, functions, as well as lethality
The Inventory of Statements about Self-injury (ISAS) [59]	NSSI history, methods, frequency, and functions
Nonsuicidal Self-injury Assessment Tool (NSSI-AT) [60]	NSSI history, methods, frequency, functions, addictive qualities, context of NSSI (e.g., social setting, routines), NSSI treatment experiences
*Interviews*	
Self-Injurious Thoughts and Behaviors Interview (SITBI) [61]	NSSI history, methods, frequency, functions as well as lethality. Also assesses suicide thoughts and behaviors
Suicide Attempt Self-Injury Interview (SASII) [62]	NSSI history, methods, frequency, functions as well as lethality. Also assesses suicide thoughts and behaviors

#### Risk assessment protocols

Investigator teams will need to determine their specific criteria for gauging level of risk and to ensure that all staff are qualified and capable of assisting in timely review of questionnaire data. Criteria for risk will vary and in part be determined by the research context and proximity to data. With the exception of studies in which participants remain anonymous, risk criteria should be determined by the researchers beforehand and clearly outlined. A risk assessment protocol should at the very least contain the following elements, described in more detail below: screening for risk, reviewing the evidence, and deciding when and how to intervene.

##### Screening for risk across various study designs

Screening of questionnaire responses should take place within 24 h of data collection when at all possible, and immediately if data collection is conducted in-person. Items to screen for should, at the very least, include: suicidal ideation (i.e., passive/active: thoughts of death, thoughts of killing oneself), depression level, and NSSI behaviors (i.e., frequency, form, and timing).

In the context of face-to-face research designs, it may be feasible to review data within 24 h and determine level of risk. Principal investigators should ensure that research staff are trained to identify questionnaire items that correspond with known risk factors for suicide. For example, suicide items might be flagged such that researchers can check the status of these items daily. Often, research on NSSI is web-based and/or involves screening large samples of participants completing anonymous questionnaires. In such cases, it would be nearly impossible to manually identify an individual who presents with a number of risk factors. It is also common that data collected via the web or other large-scale survey design is not always available in real time, so even if tracing an individual were possible, it would be unlikely to occur in a timely fashion. In order to manage liability issues related to collecting sensitive information on safety and risk, researchers may consider avoiding assessment of *current* (i.e., past 24 h) NSSI intentions and behaviors in their research design, instead focusing on recent and past experiences.

Finally, online data is commonly collected anonymously, thereby hindering researchers from being able to screen and respond to high-risk cases. This may potentially reduce legal responsibilities, but perhaps does not reduce ethical and moral considerations. Researchers might opt to include a link on every survey page for local or immediate mental health resources should someone feel triggered by survey content. Online data collection tools can be set up so that an email alert to study staff is directly tied to certain item responses. These pre-selected items, if chosen by participants, could prompt automatically generated responses to individuals and they can be provided with suicide prevention resources. As mentioned previously, some researchers have also begun including the use of a “distract button” which allows participants to click at any point during the survey, taking them to a non-emotional webpage (e.g., WSJ.com) for a chance to regroup, and then return to complete the survey.

##### Reviewing identified cases

 Particularly in the case of face-to-face research, the above information should be reviewed by senior staff or project principal investigator in the context of information from other useful questionnaire items, such as substance abuse, history of abuse, recent losses or other stressful life events, and lack of social support in determining risk. Both distal risk factors (e.g., history of past suicide attempt) and the current state of the individual should be taken into account when assessing suicide risk [22]. Based upon review of the suicide [23] and NSSI literature [16, 24], researchers may want to consider establishing risk ratings along with specific descriptions of each in order to assist study staff in consistent and reliable evaluation of cases involving face-to-face research. For example, Joiner and colleagues (p. 451) provide examples of suicide risk ratings on a continuum from nonexistent (no identifiable suicidal symptoms, no past history of suicide attempt, and no or few other risk factors) to extreme (a multiple attempter with severe symptoms of the resolved plans and preparation factor and two or more other risk factors), along with recommendations for action (e.g., hospitalization, safety plan) [23]. The literature on risk factors for suicide is extensive, and a comprehensive review of this area is beyond the scope of this manuscript; however, there are numerous excellent reviews on this topic [25–27].

Other risk management protocols include the University of Washington Risk Assessment Protocol (UWRAP) [28], which includes instructions for managing risk during and following assessments with suicidal and other highly distressed patients, and the Linehan Risk Assessment and Management Protocol (LRAMP) [29], which can serve as a guide for suicide risk assessment documentation. In addition, researchers may consider including the Columbia Suicide Severity Rating Scale (C-SSRS) [30], a screening tool for suicidal ideation and behavior, in study protocols involving face-to-face research where suicidal ideation and behavior are of particular concern.

#### Deciding whether to break confidentiality and how to intervene

The decision of whether to break confidentiality is a complicated one. It demands consideration of what is ethically required, what is mandated by IRB requirements, what is feasible given study design and constraints, and what is clinically indicated/warranted for a particular participant. Often these interests overlap, although not always. Moreover, breaking confidentiality cannot be assumed to be beneficial for all involved. Indeed, breaking confidentiality can cause harm to the adolescent and the relationship between adolescent and researcher or even pose harm to the adolescent through exacerbating unhealthy family interactions in instances where parents are alerted to a behavior or episode about which they did not know. Even though researchers (particularly those conducting face-to-face research) may view their role as highly transient and largely inconsequential in the life of her/his subject, adolescent participants are likely to view researchers as also in a therapeutic role, if only temporarily, because of the personal and sensitive nature of the topic. Because of this, breaches in confidentiality can be perceived as a betrayal. In these cases, it is important to emphasize that the pattern of scores on measures used to assess risk for other concerning behaviors suggest that the participant would benefit from making an appointment to see their mental or physical health provider.

If researchers have concerns about risk of imminent suicidal behavior, psychosis, experience of physical or sexual abuse, or risk to another person, then they have a duty to break confidentiality and seek support for the participant and any others involved. While various study designs and populations will necessitate different levels of involvement by trained clinicians, including a trained mental health professional as a member of the research team or engaging one as an on-call resource is recommended as a strategy for dealing with these uncommon occurrences across most NSSI studies. For instance, studies involving face-to-face contact, multiple visits or treatment sessions, or involving inclusion of content meant to induce an altered emotional state may particularly benefit by including a trained clinician. Studies in which data is collected anonymously would offer an exception to this recommendation. Inclusion of a trained mental health professional will also help to offer the IRB assurance that steps have been taken to anticipate any emergent clinical issues.

Conditions for breaching confidentiality (or for eliciting study team discussion of this) should be clearly articulated in advance of study execution. Ideally, cases which may trigger breaches of confidentiality will be considered by multiple study team members, according to the agreed upon protocol, prior to the breach, but this may not be possible in all cases because of study design. In cases where a breach is warranted, participants have the right to understand why this is the case and what they can expect to happen next. This is particularly important for adolescents who, by virtue of their developmental stage, may already be struggling with a sense of low autonomy and power.

In cases where red flags for confidentiality breach are present, the investigator and/or study team will need to consider the unique contextual factors at play in each case. The presence of some factors, for example, may mitigate the need for a breach in confidentiality. These include, but are not limited to: the participant is already in therapy and his/her therapist is aware of suicidal tendencies; the participant exhibits only passive ideation (e.g., thoughts of death, as opposed to thoughts of killing oneself); and/or there are no suicide plans. It is important to note that research participants reporting a history of NSSI do not automatically necessitate imminent risk and disclosure to their parents. In fact, in the absence of imminent risk for suicide, it is unlikely that confidentiality will be breached when a research participant endorses engagement in NSSI. Rather, features of NSSI behaviors (e.g., frequency, form, recency) may be conceptualized as possible risk factors for suicide; in other words, endorsement of NSSI may lead researchers to consider performing a more thorough suicide risk assessment if possible. Research indicates that a history of 20 or more lifetime NSSI incidents is associated with significantly greater risk of suicide attempts among young adults [16]. Furthermore, latent class analysis of young adults who self-injure found that a high-severity NSSI group (the ones that would most likely be considered at imminent risk) were those that also reported higher numbers of NSSI incidents than the other NSSI groups. In addition, they used more than three forms of injury that tended to be more severe, and thus were capable of causing a high degree of tissue damage and were more likely to be life-threatening in nature [16]. This higher risk group was also more likely to report current NSSI and suicidal ideation and behavior. Clearly the timing and severity of NSSI should be evaluated as potential “red flags” that may be associated with elevated suicide risk.

When participants who are under the required age to consent are determined to be at imminent risk, contacting parents/guardians is most often a first step. If a phone call to parents is warranted, this may be more seamlessly accomplished given that a relationship is likely to have already been established at the time of obtaining parental consent. If this is contraindicated because of poor guardian–child relationships (e.g., when disclosing to a parent may increase risk of suicide), direct contact with an adolescent’s therapist, general practitioner, or other local clinic or clinical support is indicated. Prinstein and Helms [10] noted that they do not attempt to conduct an additional clinical assessment with youth prior to contacting parents because research staff do not have a therapeutic relationship with the participant, which limits validity of the risk assessment, and because only in rare circumstances have they received information that changed their decision to notify parents.

Important points to note during disclosure to parents include: explaining that the study measures are not clinical instruments and thus cannot be used to detect future risk with absolute certainty; expressing concern about their child’s responses to specific items, reinforcing that the safety of their child is of primary importance; assessing whether this information is a surprise to them and whether their child is already in treatment; helping them to think about how to get a psychological evaluation of their child and encouraging them to do so; reminding them that this was hard for their child to disclose and recommending not being punitive or awkward with their child about this issue [10].

Certain research contexts may lend themselves to this type of intervention, including academic medical centers, psychiatric hospitals, and mental health facilities. If a researcher believes that a study participant is at imminent risk for suicide, they should consider immediate evaluation for psychiatric hospitalization. Regardless of risk level, researchers should always document the risk factors and associated decisions to break confidentiality thoroughly and carefully.

### Iatrogenic effects in NSSI research among youth

#### Is there risk associated with participating in NSSI research?

It is important to assess risk to potential study participants as risk-related variables can affect study design, including choice of methods, research participants, and research setting [4]. This is guided by the overarching question: will the particular methods involved in the research, or questions asked (i.e., about NSSI, suicide) exacerbate participants’ symptoms or cause undue physical or psychological distress? In some instances, the experience of psychological distress (e.g., brief induction of negative mood) may be acceptable, provided that it can be mitigated (e.g., mood is brought back to baseline) and that the benefits of the research outweigh the risks.

Indeed, IRBs may express concerns about the impact of NSSI questions and the aspect of iatrogenic risk; that is, whether by virtue of asking NSSI questions, researchers will provoke NSSI thoughts and behavior in young participants. These concerns may be especially relevant in the case of research conducted with anonymous participants or in the case of research devoid of direct interaction between researcher(s) and participant(s), such as in the case of online studies. To this end, several efforts have been made to investigate the iatrogenic risk of questions pertinent to NSSI.

In a recent study involving almost 850 young adults, participants were randomly assigned to one of two conditions [31]. The first was an experimental condition (n = 439) in which individuals were presented with questions assessing NSSI; the second was a control condition, in which these questions were not presented. The impact of asking about NSSI was then examined immediately (with pre and post-measures) and again 3 weeks later by assessing for NSSI behavior and urges. Findings indicated that responding to detailed NSSI questions did not yield significant changes in NSSI behavior or urges compared to the control group; indeed, evidence of an iatrogenic effect of NSSI questions was unsupported both immediately and 3 weeks after initially assessing NSSI. Interestingly, these findings were consistent irrespective of the severity of NSSI. These findings mirror those for suicide [32, 33]. Indeed, researchers have demonstrated that asking about suicide does not increase suicidal ideation or distress, even after accounting for suicide risk factors (e.g., depressive symptoms, substance use, past suicide attempts).

In fact, there are also findings suggesting that taking part in NSSI research may have benefits to participants. For example, Whitlock and Pietrusza [34] examined the experiences of those who take part in NSSI research; a paucity of individuals reported that questions about NSSI impacted them negatively. Rather, many noted that there were benefits to participation, namely enhanced self-reflection and, in some cases, disclosure and help-seeking intentions. Similarly, Muehlenkamp and colleagues [31] found that NSSI research participants indicated willingness to take part in NSSI research again and liked contributing to science as they believed the research was conducted for a good cause and they felt good about taking part as a result of this. Future research should further explore the potential benefits for individuals who engage in NSSI of taking part in NSSI research. Doing so is conducive to providing a fuller picture of the manner by which participants are differentially affected by different forms of NSSI research. Taking these and the above findings together, it seems that there is little empirical support for the iatrogenic risk of asking about NSSI.

#### Related concerns about iatrogenic effects

In addition to the impact of NSSI questions, IRBs may also express concerns that other research methods increase risk for NSSI or psychological distress. For example, some research involves showing images of NSSI to participants [35, 36]. Exposure to NSSI images, particularly those that are graphic in nature (e.g., photographs of NSSI), may be triggering to some individuals who self-injure [37–39]. Thus, on the one hand, there may be merit to concerns regarding the effect of exposure to these images. On the other hand, it cannot be assumed that all individuals who self-injure are impacted by NSSI images in the same manner. Indeed, some individuals may not be adversely affected by viewing NSSI images; some may even report that seeing NSSI images helps to curtail future NSSI urges and distress [37, 40]. Irrespective of how individuals are impacted by NSSI imagery, it is important to minimize how individuals may be affected within research contexts involving use of such imagery. Other research methods such as those investigating processes thought to be involved in NSSI (e.g., cognitive or emotional factors) may also raise concerns from IRBs. For instance, induction of negative affect through computer or lab-based tasks may have IRBs concerned about participant well-being. Several studies have used these [41, 42]. For example, Arbuthnott and colleagues [41] repeatedly induced rumination in a sample of undergraduate students using an online task; many of the participants had a history of NSSI. When conducting research in which psychological distress may be induced, it is important that the benefits of the research outweigh potential for psychological harm to participants. Likewise, it is imperative that safeguards be put in place to assess for and reduce this potential. We present a number of strategies for researchers to employ in these various contexts in the following section.

### Recommendations to mitigate risk

#### Provision of NSSI resources

It is recommended that when NSSI questions are asked in any research context, that participants be given NSSI resources (e.g., helpful books, websites, coping tools) in tandem with standard debriefing forms. When the research is remote, with little to no direct contact with research participants, it may be important to provide NSSI resources at all times throughout the study. For example, when conducting online research, having a hyperlink to resources on all pages of the study website may help to ensure participants can readily access resources at all times. As not all resources available are necessarily reliable [43], we provide a list of helpful resources for those engaging in NSSI in Table 2.

**Table 2:** Recommended NSSI resources for research participants

Resource name	Description
Websites	
Self-injury Outreach and Support (SiOS) www.sioutreach.org	Offers information for individuals who self-injure, including: general information, a series of coping guides, a platform to read and submit recovery-based stories; also includes guides for parents, teachers, mental health and medical professionals
Cornell Research Program for Self-injurious Behavior (CRPSIB) www.crpsib.edu	Offers information for those who self-injury, including those who self-injure, parents and teachers. Also offers research publications, NSSI factsheets, and video presentations on treatment
SAFE Alternatives www.selfinjury.com	Admission, treatment and referral information; resources; moderated blog; materials for mental health professionals

#### Elevating mood

When there are concerns that particular research approaches may produce psychological distress, mood-elevating activities can be beneficial near the end of a study. Doing so may help to ensure participants do not leave a study distressed, especially if this is determined when conducting mood checks. For example, in the study cited above, in which participants took part in consecutive rumination induction tasks, the researchers assessed mood pursuant to each rumination induction and then engaged all participants in a mood augmentation task at the end of the study [41]. Specifically, participants watched a nature video. Exposure to nature has been shown to be an especially effective manner by which to restore emotional states and may have salience for those who experience mental health difficulties [44]. At the end of this study, participants’ moods were actually higher than they were at the start of the study [41]. These approaches can be used in online contexts and in lab settings. As participants may prefer different techniques to reduce distress and improve their mood, it may be helpful, when possible (e.g., when there is direct contact with participants) to ask participants at the outset of a study to indicate what might help them should they become distressed. If feasible, these techniques can then be used by the researchers at the end of a study.

#### Use of distract buttons

While current evidence suggests that there is no iatrogenic effect associated with asking about NSSI, online research (in which there is typically no direct contact between researchers and participants) is unique and particular methods to ensure participant well-being may be needed. For instance, it is conceivable that although NSSI questions may not evoke urges to self-injure, at least some individuals may experience discomfort at some point during their participation in a study. Indeed, in most study protocols examining any kind of mental health difficulty, IRBs suggest that participants be informed that certain questions may be upsetting or difficult. Accordingly, we suggest that distract buttons be used when conducting online NSSI research. Moreover, many IRBs may still have trepidation regarding the use of NSSI questions. The use of a distract button, coupled with the provision of resources noted earlier, may help to allay these concerns.

### Professional competency

By “professional competence” we mean the ability to appropriately apply the combination of knowledge, experience and judgment, which is built on a foundation of skills, knowledge and moral development [45, 46]. Traditionally, risk assessments in research have focused on mitigating the risk to research participants. While this is of course important, there is also a need to focus on the risk to researchers. This dual focus relies on ensuring that the researchers involved have the professional competence both to look after their participants *and* themselves.

#### Ensuring participant and researcher safety

When planning research, duty of care towards participants has prime importance in the research proposal and in the ethical review process. However, duty of care towards researchers and research staff is often limited to a focus on physical safety at the expense of the potential emotional impact the research could have. Indeed, in their review of risk to well-being, Bloor and colleagues [47] concluded that while researchers are good at looking after participant safety, they were far less attentive to their own. Similarly, Moncur [48] noted that consideration for the well-being of the researcher was only formalized in two out of eleven institutions that participated in her research. This is an important omission, particularly for researchers who engage in qualitative or interview-based research who are essentially “entering the life-world of participants” [49]. In fact, there is evidence to suggest that engaging in qualitative research on sensitive topics has the potential to pose a threat to researchers’ well-being, particularly if they have strong feelings, or have some experience of the issue being researched [50]. In addition, we should not forget that those whom researchers employ to transcribe interviews are also subject to the same emotional response; while they type, they too hear the stories as told by the participants and so may also experience an impact on their emotional well-being [51]. By way of example, the following quote from Bahn and Weatherill [52] illustrates the impact on researchers of hearing participants’ difficult stories:

*What do you do with all this stuff in your head? There is the stuff that is used for the research, and then the stuff that ends up on the cutting room floor (and swims on your head in your quiet moments). No matter how experienced you are, it has to go somewhere or I think I would carry these people around with me for a long time (p. 27).*

One means for addressing this is to ensure that the research team has regular debriefing and supervision sessions. As Moncur notes, access to support combined with opportunities for reflection is an integral part of professional practice in health-related professions, such as psychology. Engaging in reflective practice within supervision facilitates the ability to take a step back and analyze the experience. In the UK, the British Psychological Society (BPS) Code of Ethics and Conduct makes it clear that psychologists should engage in regular supervision sessions, particularly when “circumstances begin to challenge their scientific or professional expertise” (p. 16) [53]. Engaging in reflective supervision would ensure that researchers understands their emotional response to the data, protects the researchers’ emotional well-being, and enables researchers to explicitly reflect on and factor into the analysis their responses to the data. Gaining insight into one’s reactions to the data will also facilitate the researcher’s ability to develop what Walsh calls a “low key dispassionate demeanor” and what Kettlewell describes as respectful curiosity [54, 55]. Essentially this is an ability to be interested in NSSI while maintaining a non-judgmental manner. This may be especially important for interview-based research, but the use of a respectfully curious tone should also be evident in the wording of any quantitative surveys as well.

One important point to consider when conducting research with individuals who engage in NSSI is that unlicensed staff may be collecting study data (i.e., graduate and undergraduate students, research assistants, clinical evaluators, post-doctoral fellows). Therefore, all staff should be properly trained (or supported by trained staff) in defining, assessing and addressing potential suicide risk. At the same time, it is best practice to have a licensed clinician on call during data collection, in order to provide clinical judgment in cases that require a breach in confidentiality or incident reporting [10].

### Areas in need of further discussion and research

Despite the areas of agreement and consensus in NSSI research, there exist a number of areas in which variation in approach and sensibilities remain rather broad and inconsistent from one researcher to another. One of the primary areas affected by this diversity of opinion and approach is the conditions under which confidentiality can be or should be breached, but it is not the only area. Anticipating and mitigating ways in which studies may inadvertently contribute to iatrogenic effects, how and under what conditions clinical staff or members of the study team need to be available, and methods for accommodating international variation are other areas in which the field as a whole would benefit from discussion and higher degrees of agreement.

Consider breach of confidentiality as a case in point. Myriad factors, linked to study design, participant history and context, and study team approach and perspective influence decisions in this arena. Some researchers, for example, feel strongly that all NSSI research, even if web-based and spanning large numbers of participants residing in a variety of geographic areas, should collect names and contact information for all respondents and should review all NSSI cases for possible imminent suicidal risk. Others maintain that this kind of surveillance, review, and possible intervention is not viable in this kind of research. They also point out that asking for contact information can reduce honesty of responses; one of the clear benefits of web-based research. This is just one example of many which highlight the divergent perspectives, opinions and approaches among even well established NSSI researchers related to when, where, and under what conditions confidentiality should be breached. As a result, we are unable to offer specific “best practices” in this arena.

Fortunately, the NSSI research community faces a unique and invigorating opportunity to both take stock of all we have collectively learned and can pass on to new researchers in this area and to simultaneously identify areas in which we would benefit from more organized discussion. Toward this end, we have hoped to clearly lay out the former with this publication, and aimed to synthesize in concise and clear ways lessons learned from over a decade of research in this area. We leave NSSI-dedicated scholars with a set of questions in need of discussion and some degree of consensus:

- What specific conditions, behaviors, or other risk indicators, when present, should trigger “breach of confidentiality” protocols? For example, prospective studies of suicide risk suggest that NSSI is a strong risk factor for suicidal behavior, however, these studies typically use a long-term follow-up period [56]. Therefore, further research is needed to ascertain whether specific characteristics of NSSI (e.g., medical severity, frequency, recency) predict *imminent* or short-term risk for suicidal behavior.
- How should study design interact with the above list of conditions, behaviors, or other risk indicators? More specifically, how should (a) a list of conditions, behaviors, and other risk indicators and (b) breach of confidentiality protocols be altered based on study design?
- What factors contribute to elevated distress and NSSI urges pursuant to viewing or accessing NSSI imagery and other NSSI-themed content (e.g., NSSI text or narratives) for some individuals but not others? Relatedly, which individuals are more vulnerable to these risks?
- Are there iatrogenic risks associated with NSSI research in which NSSI is simulated (e.g., use of a cold-pressor task) or in which distress is induced? This line of empirical inquiry would also help to understand whether findings from research examining the iatrogenic effect of asking about NSSI can be generalized to other study types [31].
- How do emerging methodologies used to represent proxies to NSSI impact participants? For example, a recent study involved giving incisions to the forearm of participants following an induction of stress [57]. What are the perceived benefits of this line of work? Do the perceived benefits outweigh and justify the potential impact on participants? Do these and other approaches represent a valid parallel to self-inflicted NSSI? Should these methods be used in the future, it will be critical to understand whether they have an iatrogenic effect or whether they adversely affect participants in other manners (e.g., psychological distress).
- What exacerbating (e.g., dangerous family environment) or mitigating (e.g., already in therapy) factors should also be collected in each case and what are best practices for weighting these in service of a final decision regarding risk and breach of confidentiality?
- What are the range of possible breach of confidentiality protocols in use by NSSI researchers and how might these be distilled into a set of best practices in development of protocols which also reflect study design allowances and limitations?
- How can we proactively develop strategies that allow for being mindful of the wider international context? Sharing ideas and knowledge among researchers from different countries and backgrounds will further collaboration, extend our thinking on issues that many of us are pursuing from various angles, and allow for development of a richer understanding of ‘other’ perspectives. This could involve establishing research advisory groups that have representatives from more than one country. The International Society for the Study of Self-Injury (ISSS) is positioned to take a leadership role in establishing mechanisms that facilitate this international discussion and collaboration.
- Similarly, how can we continue to proactively encourage dialogue between clinicians who provide treatment to those who self-injure, and researchers who study, sometimes in minute detail, NSSI and its related processes? There is much to be learned in the dialogue between clinicians and researchers, and this will surely help to inform future conversations relating to the ethical conduct of NSSI research.

It is our hope that these questions will serve as the foundation for future conversation among NSSI researchers at professional conferences and through other professional channels. Such conversation and concomitant systematic assessment of the results, would advance collective capacity to identify a set of best practices which could then be systematically utilized and empirically/experimentally tested (in cases where significant discrepancy in approach is revealed).

## Conclusions

Conducting NSSI focused research with adolescents raises a myriad of complex issues. Knowledge of clinical and research issues, and the associated ethical issues, will assist in the development of effective guidelines that researchers may use for management of these issues in youth engaging in NSSI and other high-risk behaviors. This manuscript’s intent is to offer guidance and recommendations on how to navigate these issues. While this paper has aimed to clarify and be prescriptive, we have also highlighted important areas of ambiguity and where discussion and research can help to shed light. As more research on NSSI is conducted, it will be important for researchers to remain informed on the various evolving ethical issues that may arise. In light of the emerging complexity of the field and topic, we encourage discussion of these topics and consensus building within the research community.

## Endnote

^a^A Certificate of Confidentiality is a U.S.-based, National Institutes of Health-specific protection that authorizes researchers who are engaged in biomedical, clinical, behavioral and other research to protect the privacy of individuals who are participants in sensitive research activities. The very nature of research investigating NSSI and suicide suggests that U.S.-based researchers may want to consider applying for a Certificate of Confidentiality on NIH-funded research projects. For further information, please see http://grants.nih.gov/grants/policy/coc/background.htm

## Abbreviations

NSSI: non-suicidal self-injury
IRB: Institutional Review Board
